# Dentistry and Bioterrorism: A Legitimate Threat

**DOI:** 10.7759/cureus.59958

**Published:** 2024-05-09

**Authors:** Supreet Kaur, Chandrika Karwasra, Shreya Poduval, Nityanand Shetty, Prachi Gholap, Prasad Mhaske

**Affiliations:** 1 Periodontics, Sri Guru Ram Das Institute of Dental Sciences and Research, Amritsar, IND; 2 Dental Surgery, Baba Jaswant Singh Dental College, Hospital & Research Institute, Ludhiana, IND; 3 General Dentistry, Mathrusri Ramabai (MR) Ambedkar Dental College, Bengaluru, IND; 4 Orthodontics and Dentofacial Orthopaedics, Bharati Vidyapeeth (Deemed to be University) Dental College and Hospital, Navi Mumbai, IND; 5 Prosthodontics, Bharati Vidyapeeth (Deemed to be University) Dental College and Hospital, Navi Mumbai, IND

**Keywords:** outbreak-preparedness, public health emergency of international concern, prophylaxis, public health surveillance, bacterial and viral agents, public health concern, bioterrorism, clinical dentistry

## Abstract

Bioterrorism involves the deliberate release of harmful biological agents, such as bacteria and viruses, aimed at causing mass casualties within a population. Often referred to as "poor man’s nuclear weapons," chemical and biological weapons pose a significant threat due to their potential for mass destruction. Detecting and preventing bioterrorist attacks is challenging, making them one of the most feared scenarios. Dentistry plays a crucial role in responding to bioterrorism and other catastrophic events, leveraging its personnel and facilities effectively. This paper explores the signs and symptoms of biological agents used in mass destruction, as well as the oral and dental manifestations of both naturally occurring and bioengineered infectious agents.

Furthermore, the article stresses the importance of countermeasures against bioterrorism, including deterrence, prevention, surveillance, medical management, and training. Emphasis is placed on implementing surveillance systems, bolstering medical readiness, and conducting training programs to effectively detect, assess, and respond to bioterrorism threats. Ultimately, the article underscores the critical role of dentists and healthcare professionals in collaborative efforts to mitigate the impacts of bioterrorism through proactive measures.

## Introduction and background

Dentists, long recognized for their role in forensic odontology during mass disasters, now find themselves as critical first responders in today's world, particularly in the face of terrorist attacks involving explosives, chemicals, biological agents, radiation, or nuclear materials [1]. Their extensive training equips them to play a crucial role in early detection and response to bioterrorist events [2]. As traditional medical systems may become overwhelmed during such crises, the dental community emerges as a vital resource, offering expertise in patient care, infection control, and emergency medical support [3].

In a major bioterrorist attack, dental professionals may be tasked with diverse responsibilities ranging from education and risk communication to diagnosis, treatment, and even forensic dentistry [4]. Their involvement extends to medication distribution, decontamination, sample collection, and surveillance, underscoring their multifaceted contribution to emergency response efforts [5]. This article sheds light on bioterrorism and its causative agents, setting the stage for subsequent discussions on the indispensable role dentists can play in mitigating the impact of such events.

## Review

Methodology

This review article involved an assessment of published studies providing a comprehensive discussion of bioterrorism and the role of dentists as healthcare providers in curbing the spread of the same. Multiple databases, including PubMed, Web of Science, and Google Scholar, were utilized to collect the most relevant articles on this subject. The search used several terms such as “dentistry”, “bioterrorism”, “bacterial and viral agents”, “prophylaxis”, “surveillance”, “COVID-19”, and “public health concerns”. By using this method, all the studies discussing bioterrorism, its history, causes, biological agents, and countermeasures for responding to the crisis were included in the study. Studies with poor methodological quality, outdated data, or insufficient data were excluded. The most relevant articles were selected and included in this review. This review ultimately involved 33 papers related to bioterrorism and dentistry from 1977 to 2021.

What is bioterrorism?

Bioterrorism involves the intentional misuse or threat of microorganisms or toxins from living organisms to cause harm or illness in humans, animals, or plants, typically intending to instill fear or coerce governments or societies to comply with certain political, religious, or ideological objectives. While major metropolitan areas, urban centers, and regions near international borders are primary targets, no part of a country can be deemed invulnerable [6].

Why bioterrorism?

Bioterrorism poses a significant threat for multiple reasons. Many foresee the upcoming century as the "century of biology." Yet, the swift and profound advancements in genetic modifications, bio-molecular engineering, and enhanced bio-production technologies may lower the barriers that previously hindered terrorists from acquiring biological weapons [7]. Biological weapons provide a cost-effective and immensely destructive option compared to chemical and nuclear arms. Termed as "poor man's nuclear weapons," they are relatively simple to manufacture, widening their accessibility to various adversaries. Furthermore, the concealment, transportation, and spreading of deadly amounts of infectious agents pose considerable obstacles to detection and containment efforts. Once unleashed, these agents can spread over vast areas, with symptoms often appearing days or weeks later, complicating both identification and response strategies.

Moreover, certain biological agents possess a concerning ability for secondary transmission, allowing them to spread from one infected individual to others, dramatically increasing the potential extent of damage. Additionally, the environmental repercussions of bioterrorism can be significant. Apart from the immediate physical consequences, the deployment of biological weapons can trigger widespread panic and fear among populations, heightening the chaos and disruption caused by these attacks.

History of biological warfare

Biological warfare has ancient roots, evidenced by early civilizations using toxins from plants and animals for hunting and combat. In the sixth century BC, the Assyrians poisoned an enemy well with rye ergot, causing convulsions upon ingestion [8]. Similarly, during the siege of Caffa in the 14th century, attackers catapulted plague victims' corpses over city walls, sparking a devastating plague outbreak known as the ‘Black Death’ Pandemic [9]. Additionally, during the French and Indian wars in 1520, British forces purportedly distributed smallpox-laden blankets to Native American tribes loyal to the French, resulting in widespread infections [10].

In more recent history, in 1915, Dr. Anton Dilger, backed by the German government, infected thousands of livestock destined for Allied troops in Europe with Bacillus anthracis and Pseudomonas mallei [11]. Sympathetic dockworkers in Baltimore were provided with the agents and an inoculation device to infect thousands of animals intended for military use. Despite the Biological and Toxin Weapons Convention (BTWC) signed in 1972, prohibiting the stockpiling of biological agents for military purposes, some nations such as the former Soviet Union and Iraq persisted in intensifying biological weapon production [8].

Bioterrorism: a current legitimate threat

In the autumn of 2001, the dissemination of weapons-grade Bacillus anthracis spores via the U.S. postal system drew global scrutiny regarding the potential use of biological weapons [5]. The spores, smaller than the pores in paper envelopes, could readily escape during mail processing [6]. This incident has heightened apprehension among government authorities and the healthcare sector regarding the deliberate release of deadly infectious agents [12].

Detection of a bioterrorism event

Clues Suggesting a Bioweapon Release

A sudden surge in disease cases within a short timeframe, often spanning mere days or hours, leading to a substantial influx of patients at once, characterizes the first indicator of a potential epidemic [13]. What distinguishes this outbreak is the alarming fact that previously healthy individuals are among those affected. The seriousness of the illness manifests in elevated rates of both morbidity and mortality, characterized by prevalent symptoms like severe pneumonia, dyspnea, and septic shock. Heightening suspicions are any recent terrorist assertions or activities, amplifying apprehensions of intentional harm. Moreover, an unexplained upsurge in illnesses or deaths among animals, termed an epizootic, acts as another substantial indicator of a potential biothreat.

Indications of Intentional Release of a Biologic Agent

Signs indicating a potential infectious disease outbreak include unusual patterns of illness occurrence either in terms of time or location, along with the identification of individuals who attended the same public gathering or event. Moreover, healthcare providers should be vigilant for patients displaying clinical symptoms suggestive of an infectious outbreak, such as unexplained febrile illness associated with sepsis, pneumonia, respiratory failure, or rash, especially when observed in more than two patients.

Additionally, the emergence of a botulism-like syndrome characterized by flaccid muscle paralysis, particularly in otherwise healthy individuals, warrants attention. Furthermore, any deviations from the expected age distribution for common diseases, such as a rise in adult cases resembling chickenpox but potentially indicative of smallpox, should raise concerns and prompt further investigation.

Delivery mechanisms

Aerosol dissemination stands as the primary method for spreading the majority of agents, with the potential for bioengineering these agents to optimize aerosol delivery [13]. This method is favored for its ease of dispersion, resulting in the highest exposure rates among the population and offering the most efficient and contagious route of infection. The less likely method of transmission involves contamination of food or water sources, which is effective only for certain agents. Deliberate infection of a terrorist with any transmissible agent entails attempting to infect others who come into contact with the affected individual. For this strategy to succeed, the agent would need to possess a high level of infectiousness, although it may not result in widespread infection among many people.

Bioterrorism agents

**Figure 1:**
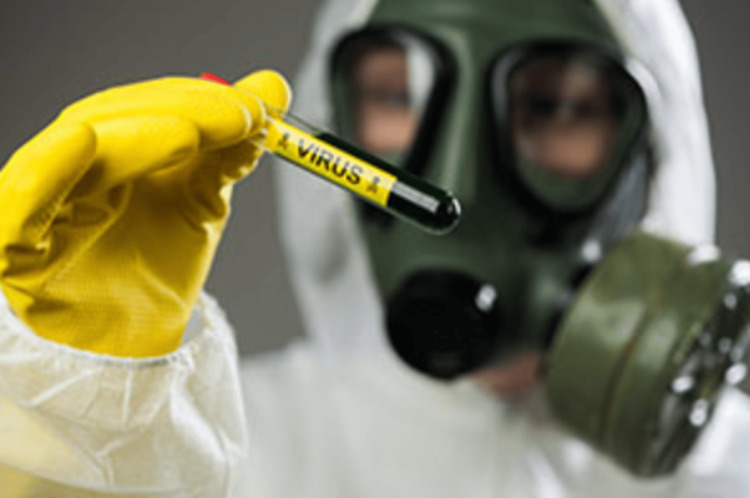
Biological weapon - virus Courtesy: https://www.thefederalcriminalattorneys.com/federal-biological-weapons-laws

While any germ, bacteria, or virus holds the potential for use, certain biological agents are considered more likely to be employed due to their accessibility to terrorists and the simplicity of their dissemination (Figure 1). The Centers for Disease Control and Prevention (CDC) have classified these agents into three categories based on these factors [14].

CDC Category A agents are top priority due to their significant national security risk, being easily transmitted and causing high mortality rates with substantial public health impacts. Notable examples include anthrax, botulism, plague, smallpox, and tularemia, demanding meticulous preparation and response planning from emergency planners and public health agencies.

In contrast, Category B agents pose a considerable risk, although they may not spread as easily as Category A agents. Despite this, they can still cause moderate illness and death rates, requiring enhanced diagnostics and surveillance for effective monitoring and control. Examples include Q fever, brucellosis, and glanders, as well as certain bacterial pathogens like Clostridium perfringens and Staph enterotoxin B.

Lastly, Category C comprises emerging pathogens that could be manipulated for bioterrorism. These agents are easily accessible and producible, potentially causing significant illness and death despite not being widely recognized. Diseases such as Nipah virus and hantavirus fall into this category, emphasizing the need for vigilant monitoring of influenza-like symptoms for early detection and response to potential bioterrorism events involving Category C agents [15].

COVID-19

In March 2020, the World Health Organization declared the outbreak of coronavirus disease 2019 (COVID-19) a global pandemic. The ongoing crisis has had an unprecedented impact not only on public health but also on the global economy, social well-being, and critical infrastructure. The COVID-19 pandemic underlines the catastrophic consequences of infectious disease events of this kind and highlights the similar effects that an act of bioterrorism could have [16].

The COVID-19 pandemic has sparked debates about its origins, with initial reports suggesting a zoonotic transfer in Wuhan, China, while alternative theories, such as a viral bioweapon or a laboratory leak, persist amidst widespread misinformation [17]. However, a closer examination reveals that neither bioweapons nor a laboratory leak is a likely explanation for the pandemic's origins.

Bioweapons, despite their potential for mass destruction, have historically been distinct from natural outbreaks in terms of scale and scope. International treaties, like the Biological Weapons Convention of 1972, have criminalized their production and stockpiling, making them highly improbable causes of the COVID-19 pandemic [18].

Similarly, concerns about a laboratory leak are challenged by scientific evidence indicating the virus's natural origins. Previous incidents of accidental releases from research facilities do not align with the genetic makeup of SARS-CoV-2, which lacks indications of human manipulation. Studies suggest that the virus likely underwent natural selection in animal hosts before being transmitted to humans, refuting notions of a laboratory-engineered pathogen [19]. This underscores the importance of evidence-based inquiry in understanding the pandemic's origins.

Anthrax

Anthrax, a severe bacterial infection caused by Bacillus anthracis endospores, possesses qualities making it an ideal bioweapon, including its odorless, colorless, and tasteless nature [8]. Its production demands minimal expertise, often utilizing equipment originally intended for vaccine production [8]. Anthrax can enter the body through skin abrasions, inhalation, or ingestion, resulting in distinct clinical presentations: cutaneous, inhalational/pulmonary, and gastrointestinal/oropharyngeal anthrax.

Cutaneous anthrax typically manifests as painless red papules that may progress to malignant pustules if untreated, with mortality rates reaching up to 20% in untreated cases and less than 1% in treated cases [20]. Inhalational anthrax, the most lethal form, leads to hemorrhagic necrotizing mediastinitis and sepsis, boasting a mortality rate of up to 95%. Unlike pneumonia, inhalational anthrax doesn't directly infect the lungs but induces severe bloodstream infection (bacteremia), disseminating to multiple organs, including the meninges, culminating in meningitis in 50% of cases [21]. The 1979 Sverdlovsk epidemic, triggered by the accidental release of 1 gram of anthrax spores, resulted in 66 human fatalities and numerous cattle deaths [21]. Despite an average symptom duration of about four days before seeking medical attention, survival post-hospitalization averaged just 1 day. Gastrointestinal anthrax, contracted through contaminated meat consumption, presents with fever, toxemia, and oral lesions, carrying mortality rates ranging from 25% to 60% [21].

Differential diagnosis involves distinguishing anthrax from common respiratory infections based on the absence of typical flu symptoms like sore throat and rhinorrhea, as well as the presence of shortness of breath, which is characteristic of anthrax [15].

Smallpox

Declared eradicated in 1980 due to concerted efforts by the World Health Organization, smallpox stands as one of the most dreadful human diseases [20,21]. Despite its eradication, the virus persists, with stockpiles of attenuated viruses for immunization existing alongside the possibility of fully active viruses being weaponized by malicious actors [22,23]. Smallpox can manifest in mild or virulent forms, with an incubation period of 7-17 days during which the patient remains asymptomatic and non-contagious [21]. The onset of symptoms includes fever, malaise, and headache, akin to influenza, followed by the appearance of a characteristic rash within 2-3 days. The rash progresses from macules to papules to vesicles and eventually to pustules, with lesions developing in the nose and mouth, releasing large amounts of the virus, and rendering the patient highly contagious [8].

Smallpox is highly infectious, with less than 10 virions or a mere 15-minute aerosol exposure being sufficient to infect a healthy individual. The disease can spread through various bodily fluids like saliva, vesicular fluid, and blood, as well as via contaminated surfaces such as hospital linens [6]. Each infected person has the potential to spread the disease to 10 or 20 others, making smallpox a significant bioterrorism threat [12].

Vaccination remains the most effective method for prevention, with the smallpox vaccine derived from the closely related Vaccinia virus. A single dose provides immunity for 5-10 years, while revaccination can extend protection for over 30 years [12]. However, vaccination is not recommended for certain groups, including immunosuppressed individuals, those with a history of eczema or dermatitis, infants under one year old, pregnant or breastfeeding women, and individuals allergic to vaccine components [12].

Plague

Plague, infamous for historical catastrophes like the "Black Death," of AD 541, 1346, and 1855, remains a serious threat today [24]. Terrorists could weaponize it by aerosolizing Yersinia pestis, causing pneumonic plague with a near 100% mortality rate. Y. pestis, transmitted by flea bites, quickly spreads through lymph nodes and the bloodstream, leading to severe pneumonia and sepsis, often resulting in death [25].

Symptoms, appearing 2-8 days post-exposure, include fever, chills, malaise, and painful swollen lymph nodes (buboes), restricting movement. Necrosis of blood vessels may cause gangrene, especially in the nose, fingers, and toes [25].

In a bioterrorist event, rapid diagnosis is challenging, with cultures taking over 24-48 hours and post-exposure vaccination ineffective. Vigilance and suspicion are crucial for identifying sudden clusters of severe pneumonia and sepsis [26].

Tularemia

Tularemia is an infection caused by Francisella tularensis, one of the most infectious pathogenic bacteria commonly found in animals, particularly rodents, rabbits, and hares [27]. Infection can occur with as few as 10 microorganisms [28]. Due to its extreme infectivity, ease of dissemination, significant potential to cause illness and death, and history of being used as a bioterror agent, F. tularensis is considered a dangerous biological weapon.

Clinical manifestations of tularemia include the sudden onset of an acute febrile illness characterized by fever, chills, headache, body aches, coryza, and sore throat. In some patients, the illness progresses to involve pharyngitis, bronchiolitis, pneumonitis, pleuritis, and hilar lymphadenitis. If left untreated, the infection can lead to complications such as sepsis and inflammatory response syndrome [28].

Oropharyngeal tularemia arises from consuming contaminated food or water, resulting in symptoms like stomatitis, exudative pharyngitis, or tonsillitis. A prominent symptom is pronounced cervical and/or retropharyngeal lymphadenopathy. Tularemia pneumonia, another significant complication, occurs when the organism is inhaled directly, with secondary spread happening through hematogenous spread.

Botulinum Toxin

**Figure 2:**
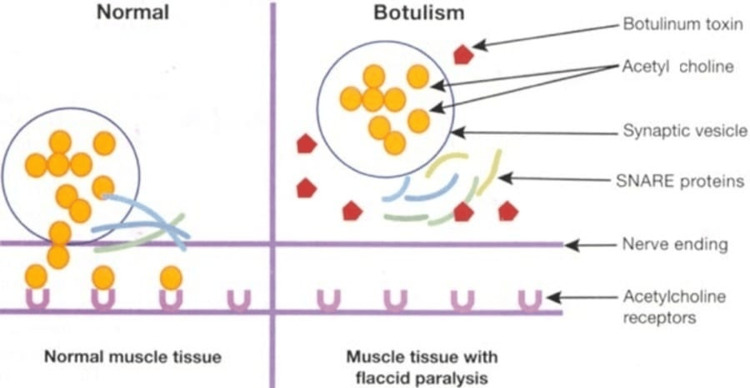
Mechanism of action of botulinum toxin Courtesy: https://www.manhattandermatologistsnyc.com/procedures/botulinum-toxin/

Botulism, stemming from Clostridium botulinum, unleashes the most potent neurotoxin known, leading to flaccid paralysis by impeding acetylcholine release (Figure 2). While typically transmitted through food, inhalation cases are plausible, yet person-to-person transmission doesn't occur. Its extreme potency, lethality, and ease of production and transportation render botulism a formidable bioweapon threat, demanding extended intensive care for affected individuals [29].

Symptoms of botulism encompass speech, vision, and swallowing difficulties, coupled with neurological manifestations like ptosis, diplopia, blurred vision, dysarthria, and dysphagia. Initial gastrointestinal distress, nausea, and vomiting may precede these symptoms. Notably, acute, afebrile, symmetric descending paralysis of facial muscles and multiple cranial nerve palsies characterize the condition, with patients remaining responsive as the paralysis targets only motor nerves [30].

The onset and severity of paralysis hinge on toxin absorption and route, with incubation periods spanning 12-24 hours after ingestion and 24-72 hours after inhalation. Additional neurological signs include loss of head control and diminished deep tendon reflexes, with death arising from airway obstruction due to respiratory and diaphragmatic muscle paralysis. Diagnosis arises from clusters of food poisoning cases, prompting botulinum toxin detection in serum, stool, or food. Prevention strategies involve vaccine development, offering immunization protection for at least one year, although routine immunization isn't currently recommended. Effective heat treatment of food minimizes risk due to the toxin's heat lability, while strict adherence to international food transport guidelines remains pivotal. In case of illness, supportive care alongside passive equine antitoxin administration is advisable.

Hemorrhagic Fever

Ebola, Marburg, and related viruses, categorized as Filoviruses and arenaviruses, are the culprits behind this disease. Typically, the incubation period spans from five to ten days. Symptoms emerge suddenly, featuring fever, myalgia, and headache. Nausea, vomiting, abdominal pain, diarrhea, chest pain, cough, and pharyngitis may also afflict patients. Around five days after the disease's onset, a maculopapular rash typically surfaces, primarily appearing on the trunk. Progression of the illness may lead to bleeding manifestations such as petechiae, ecchymoses, and hemorrhages [31].

As the disease advances, patients often encounter a range of symptoms, including fever, myalgia, and headache, followed by nausea, vomiting, abdominal pain, diarrhea, chest pain, cough, and pharyngitis. Roughly five days post-onset, a maculopapular rash typically emerges, primarily affecting the trunk. Subsequent progression may bring about bleeding manifestations like petechiae, ecchymoses, and hemorrhages [31].

Countermeasures for bioterrorism

Countermeasures against bioterrorism necessitate a comprehensive approach, as no single strategy is sufficient on its own. Key measures include the following [32].

Deterrence

The US Senate enacted the "Bioterrorism Act of 2002" to bolster biodefense, emphasizing national preparedness against bioterrorism by safeguarding drugs, food, and water from biological threats [33].

Prevention

The revised International Health Regulations, enforced in India since June 2007, aim to expedite the detection and investigation of outbreaks and other international health emergencies, facilitating collective action to contain and prevent their spread [34]. To bolster the National Disaster Response Force (NDRF), two additional battalions, each comprising 1000 personnel, have been approved. Currently, the NDRF consists of eight battalions. Half of the existing force is specialized in handling chemical, biological, radiological, and nuclear (CBRN) threats. Additionally, the National Disaster Management Authority (NDMA) has urged state governments to ensure that a portion of their state forces receive training in CBRN response [34].

Surveillance and Assessment

Timely implementation of surveillance systems by public health authorities is crucial for detecting early signs of a biological warfare attack. India's Integrated Disease Surveillance Project (IDSP), initiated in November 2004, integrates various health sectors to monitor both communicable and non-communicable diseases, including unusual clinical syndromes during public health emergencies [35].

Medical Management

Prompt administration of chemoprophylactic drugs to prevent disease spread is vital during a bioterrorism incident. This requires ensuring the availability of medicines and vaccines, identifying populations requiring chemoprophylaxis/immunoprophylaxis, and establishing mechanisms for drug/vaccine distribution through existing health infrastructure [36].

Training and Education

**Figure 3:**
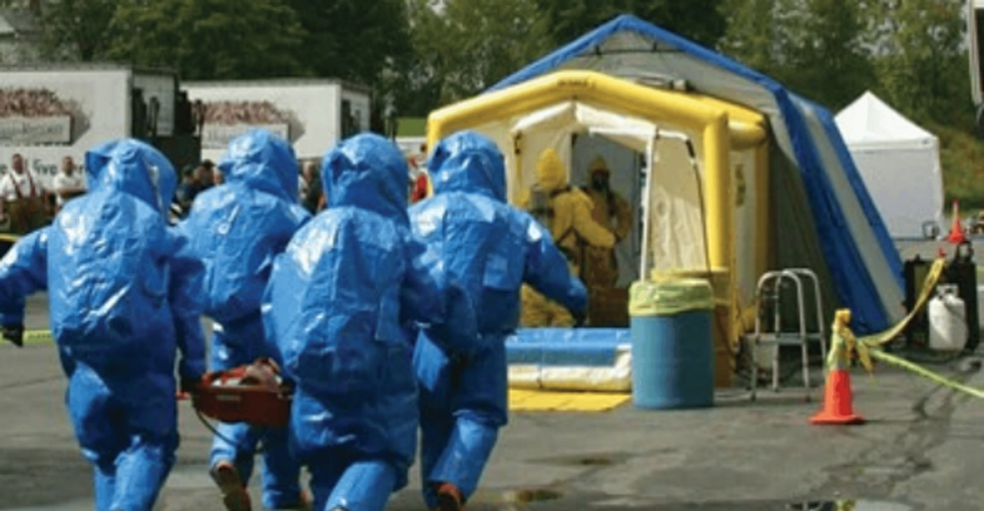
Bioterrorism - emergency training Courtesy: https://iem.com/nims-ics-training-and-bioterrorism-executive-tabletop-exercises/

Comprehensive training of medical personnel, laboratory technicians, public health officials, epidemiologists, and emergency responders is essential for effective detection, assessment, and response to bioterrorism threats. Active involvement of medical personnel in local and state government response planning is crucial for coordinated efforts (Figure 3) [37].

Role of dentists in bioterrorism

Dentists play a crucial role in preparing for and responding to bioterrorism attacks, contributing significantly to the outcome. In the event of a major bioterrorism incident, the demand for immediate healthcare could be immense, necessitating the utilization of alternate healthcare sites such as dental offices [34]. Dentists are capable of providing various essential medical interventions, including treatment for craniofacial injuries, anesthesia administration, IV line insertion, and basic life support measures. Those trained in forensic odontology can collaborate with Disaster Mortuary Operational Response Teams (DMORTs) for local surveillance and disease spread monitoring beyond the initial attack site [38].

**Figure 4:**
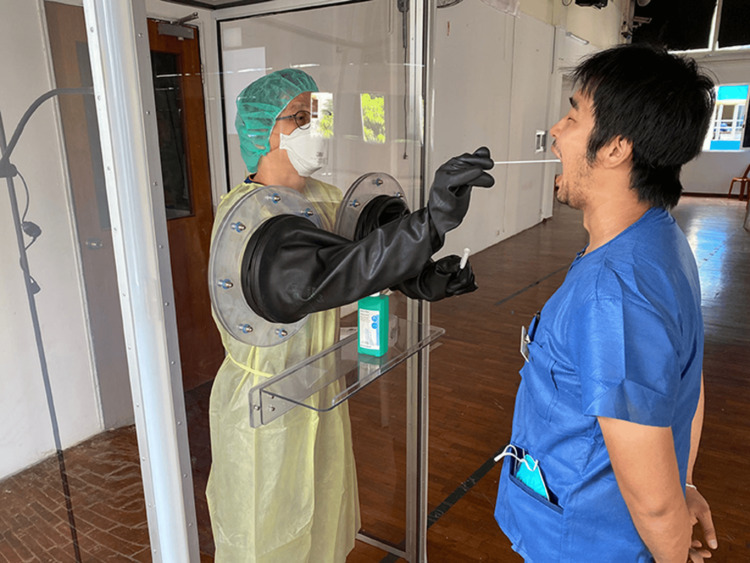
Role of dentists in bioterrorism Courtesy: https://www.frontiersin.org/articles/10.3389/fmed.2020.00566/full

The COVID-19 outbreak highlighted the importance of dentists in addressing mass casualty incidents, with licensed professionals performing diagnostic swabs and increasing screening capacity in dental clinics (Figure 4) [39]. Equipped with air and suction lines and sterilization capabilities, dental and maxillofacial offices can serve as auxiliary hospitals, aid stations, prophylaxis dispensing sites, or quarantine facilities during bioterrorism events when medical facilities are overwhelmed [39]. Oral surgeons, serving as vital communicators within the medical referral network, can undergo specialized training to become skilled responders in managing hazardous disasters, both natural and man-made [40].

Awareness of dental personnel toward bioterrorism

Dental personnel may encounter difficulties in bioterrorist situations due to limited knowledge and experience [41]. Studies indicate that both exposed and unexposed dentists demonstrate low levels of preparedness for bioterrorism events [42]. A significant proportion of dentists struggle to identify or respond effectively to such attacks. In a separate study, it was found that fewer than 15% of dentists could recognize a bioterrorism event, and less than 10% felt confident in their ability to effectively respond to a bioterrorist attack [3]. To enhance readiness and minimize harm during such incidents, dental schools should integrate pandemic and disaster preparedness training into their curricula.

## Conclusions

Biological attacks pose a serious threat due to their potential to spread rapidly, often mimicking flu symptoms and thus making detection challenging. Dental professionals, with their expertise in patient care and infection control, can play a vital role in responding to such emergencies. Incorporating bioterrorism education into dental curricula ensures all dental students are equipped with necessary knowledge and skills. Oral and maxillofacial surgeons, in particular, must be well-versed in surveillance principles and reporting procedures for potential emergencies. Mandatory training in key competencies enables them to effectively respond to bioterrorism incidents, assisting in containment efforts and participating in surveillance activities as directed by authorities.
